# Profiling protein expression in circulating tumour cells using microfluidic western blotting

**DOI:** 10.1038/ncomms14622

**Published:** 2017-03-23

**Authors:** Elly Sinkala, Elodie Sollier-Christen, Corinne Renier, Elisabet Rosàs-Canyelles, James Che, Kyra Heirich, Todd A. Duncombe, Julea Vlassakis, Kevin A. Yamauchi, Haiyan Huang, Stefanie S. Jeffrey, Amy E. Herr

**Affiliations:** 1 Department of Bioengineering, University of California, Berkeley, 308B Stanley Hall, MC 1762, Berkeley, California 94720, USA; 2 Vortex Biosciences, Inc., Menlo Park, California 94025, USA; 3 Department of Surgery, Stanford University School of Medicine, Stanford, California 94305, USA; 4 The UC Berkeley–UCSF Graduate Program in Bioengineering, University of California, Berkeley, California 94720, USA; 5 Department of Bioengineering, University of California, Los Angeles, Los Angeles, California 90095, USA; 6 Department of Statistics, University of California, Berkeley, California 94720, USA

## Abstract

Circulating tumour cells (CTCs) are rare tumour cells found in the circulatory system of certain cancer patients. The clinical and functional significance of CTCs is still under investigation. Protein profiling of CTCs would complement the recent advances in enumeration, transcriptomic and genomic characterization of these rare cells and help define their characteristics. Here we describe a microfluidic western blot for an eight-plex protein panel for individual CTCs derived from estrogen receptor-positive (ER+) breast cancer patients. The precision handling and analysis reveals a capacity to assay sparingly available patient-derived CTCs, a biophysical CTC phenotype more lysis-resistant than breast cancer cell lines, a capacity to report protein expression on a per CTC basis and two statistically distinct GAPDH subpopulations within the patient-derived CTCs. Targeted single-CTC proteomics with the capacity for archivable, multiplexed protein analysis offers a unique, complementary taxonomy for understanding CTC biology and ascertaining clinical impact.

## Abstract (web summary)

Circulating tumour cells (CTCs) are rare cells found in the blood of certain cancer patients. Here, the authors develop a cytometry tool that appends a microfluidic western blot to a CTC isolation workflow and apply it to profile a panel of proteins in single CTCs isolated from ER+ breast cancer patients.

The role of circulating tumour cells (CTCs) in cancer progression is still under investigation. CTCs are rare cells that shed from a tumour into circulation at an occurrence of 1–500 cells per 7.5 ml of blood^1^. Consequently, substantial research has focused on the isolation of CTCs by exploiting distinctive characteristics of these cancer cells (for example, surface protein expression, size and deformability)^2^,^3^,^4^,^5^. High CTC counts are associated with reduced survival rates^6^ and low responsiveness to therapies^7^. In addition, characterization of CTCs by next-generation sequencing has identified discordance in the gene expression between CTCs and their primary tumours^8^,^9^,^10^. These studies suggest that distinct CTC sub-populations may exist and contribute to metastasis. Nevertheless, although CTC enumeration and genomics provide insight, neither measurement fully describes phenotype. In fact, recent studies show weak correlation between genomics/transcriptomics and protein expression in some instances^11^,^12^,^13^,^14^.

Yet, unlike single-cell genomics and transcriptomics, advances in single-cell protein assays are lagging. Strikingly, most single-cell protein assays (for unmodified endogenous targets) are single-stage immunoassays, including enzyme-linked immunosorbent assays (with direct or sandwich readout) and immunocytochemistry, as well as newer immunoassay formats designed to improve multiplexing using spatial barcoding^15^,^16^ or mass cytometry^17^. CTC protein analyses primarily focus on surface and secreted proteins^18^,^19^. Although important, the capability to multiplex and assay a wide range of protein targets (including intracellular signalling pathways) has been limited^20^. Direct measurement of multiple proteins in single-CTCs comprises a critical complement to single-CTC transcriptomic and genomic studies, as well as enumeration.

Nevertheless, target detection by single-stage immunoassays remains constrained by the specificity and availability of immunoreagents. These limitations stymie understanding of CTC phenotype in two crucial aspects. First, single-stage immunoassays have difficulty with multiplexed measurements of surface and intracellular proteins for each single cell^21^. Immunoassays are the *de facto* standard for solid tumour and CTC classification (that is, CK+, EpCAM+ and CD45− expression). Yet, clinical immunoassays (for example, immunohistochemistry) are limited to ∼5 proteins due to spectral imaging limitations with conventional filter sets^22^,^23^ and difficulty in ‘de-staining' cells (removing antibody probes). Flow cytometry also suffers from multiplexing shortcomings, especially with intracellular protein targets. Even more importantly, neither flow cytometry nor mass cytometry can assay small numbers of CTCs, owing to cell handling losses and dead volumes^24^. Second, immunoassays cannot uniquely detect a protein if a high specificity probe is unavailable. This is of particular importance in cancer, as isoform expression is increasingly implicated in patient outcome^25^ and key isoforms do not have specific antibodies available. Although mass spectrometry can measure most protein isoforms, the analytical sensitivity is insufficient for detection of key signalling proteins with single-cell resolution^26^.

For decades, researchers have addressed single-stage immunoassay specificity limitations by prepending an upstream polyacrylamide gel electrophoresis (PAGE) protein separation to a downstream immunoassay, thus creating a two-stage assay known as western blotting. Separating proteins by molecular mass (or mobility) before the immunoassay can identify off-target, non-specific antibody binding^27^. Spatially resolving proteins by size first allows a single antibody probe to detect multiple, distinct protein forms^28^. Still, the analytical sensitivity of slab-gel western blotting requires pooling of cells to achieve detectable protein concentrations, which obscures important CTC-to-CTC protein expression level variation. To surmount this gap, we recently introduced a single-cell resolution western blot^29^ optimized for study of protein expression in each of thousands of single, cultured neural stem cells^30^ and glioblastoma cells^31^. However, the current format of the single-cell western blot requires 1000s of cells to account for cell losses when settling into the microwells.

Here we introduce a rare-cell, single-cell resolution western blot (*sc*WB) to measure a panel of proteins in single CTCs isolated from patients with primary estrogen receptor-positive (ER+) breast cancer. The rare-cell *sc*WB quantifies multiple surface and intracellular signalling proteins—in each individual CTC—allowing estimates of biological protein expression variation among CTCs, as compared with a quantitative threshold for technical variation that we establish. We show that the rare-cell *sc*WB is compatible with established CTC isolation tools, thus successfully analysing CTC populations with as few as two starting cells. In a pilot study of ER+ metastatic breast cancer patient-derived CTCs, we observe a lysis–hardy CTC phenotype and the unique capacity to normalize target protein expression by the number of CTCs analysed per assay, just one CTC per *sc*WB microwell here. The rare-cell *sc*WB offers a new approach to examining CTCs, with relevance spanning from understanding CTC biology to monitoring an individual's response to therapy.

## Results

### Workflow enables rapid protein analysis of rare cells

**Figure 1:**
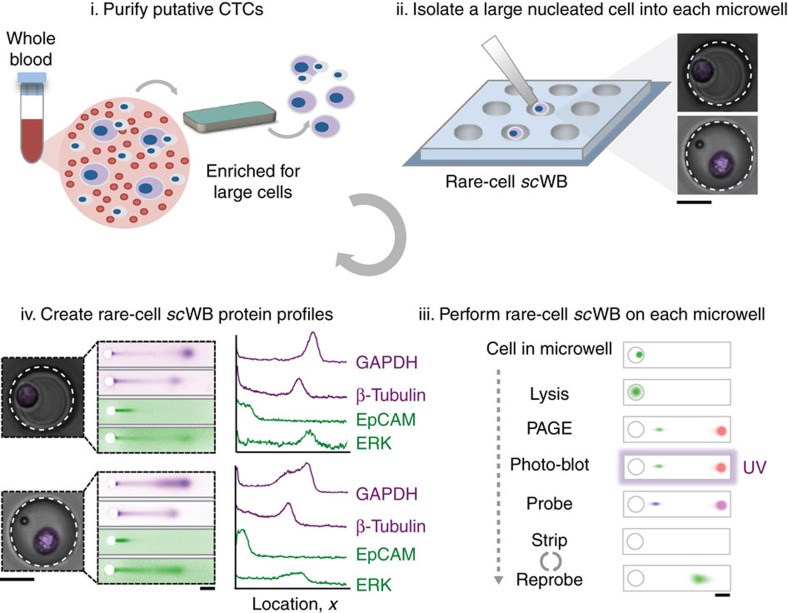
Microfluidic rare-cell workflow for multiplexed western blotting of single patient-derived CTCs. Patient-derived CTCs are: Step i: enriched from 2 to 4 ml of blood using a size- and deformability-selective microfluidic tool (Vortex HT chip)^4^ followed by Step ii: cell-enriched effluent (300 μl) is deposited directly in a mesofluidic chamber on planar *sc*WB device. Putative CTCs are visually identified using Hoescht 33342 nuclear stain, and each identified CTC is micropipetted (under microscopy) into a 50 μm diameter microwell (micrographs in inset). Step iii: after the seating of one CTC into one microwell, single-cell western blotting proceeds as in-microwell chemical CTC lysis, single-CTC protein PAGE, covalent immobilization of proteins to the gel (photo-blotting) and in-gel immunoprobing. Step iv: single-CTC lysate is analysed by western blotting and rounds of immunoprobing support the multiplexing of 12 proteins, with expression is compared among patient-derived CTCs and to spiked cell line validation studies. Scale bars, 25 μm (for the cell micrographs) and 250 μm (the separation micrographs).

We develop a microfluidic assay to measure multiple protein targets in individual CTCs. The rare-cell *sc*WB couples PAGE of single-CTC lysate with subsequent antibody probing of PAGE-resolved protein targets. The rare-cell *sc*WB assay is designed for (i) integration with existing rare-cell enrichment and isolation tools to minimize cell loss, and (ii) rapid lysis and protein separation (seconds) of isolated cell lysate to maximize local protein concentrations for detection, even with low protein copy numbers (Fig. 1). The microfluidic *sc*WB device comprises an array of 50 μm diameter microwells stippled into a 60 μm-thin polyacrylamide (PA) gel layered on a silanized microscope slide^29^. Cells are seated into the microwells, lysed and protein contents are electrophoretically injected into the surrounding gel where all steps of a western blot (PAGE, blotting and probing) are performed. The integrated design of the multi-stage assay makes rapid (seconds) assay stages possible, as is needed to minimize lysate dilution by diffusion and maintain high local protein concentrations for detection.

For analysis of rare cells, we integrate the *sc*WB with a label-free CTC collection tool that selects on cell size and deformability (Vortex chip CTC isolation technology)^32^. Although the rare-cell *sc*WB is designed as agnostic to CTC collection technology, integration with a label-free cell enrichment tool allows scrutiny of cell surface receptor identity, signalling protein levels and cell variation based on characteristics that are independent of receptor expression. Our protein panel comprises targets having accepted utility in cancer subtype classification (ER, HER2 and epidermal growth factor receptor (EGFR) oncoproteins), cancer cell identification (epithelial cell adhesion molecule (EpCAM), panCK and CK8 expression), known expression in mammalian cells (glyceraldehyde 3-phosphate dehydrogenase (GAPDH) and β-tubulin) and indicative of white blood cells (WBCs; for example, high CD45, negative for EpCAM, panCK and CK8). Profiling multiple proteins aids cancer subtype classification and may inform prognosis and therapy selection in ways not otherwise apparent from primary tumour classification^33^. Importantly, incongruences in protein expression between the primary tumour and CTCs have been observed^34^, suggesting that measurement of cancer markers in both CTCs and tumours could be informative. Overexpressed intracellular, downstream targets (that is, mammalian target of rapamycin (mTOR)^35^, extracellular signal-regulated kinase (ERK-1/2) (ref. 36) and eukaryotic translation initiation factor 4e (eIF4E)^37^) were also assayed by *sc*WB. Inhibition of intracellular signalling is an active subject of clinical trials^38^,^39^. Further, breast cancer cells have been found to have upregulated GAPDH expression, leading to interest in profiling of seemingly innocuous housekeeping proteins^40^.

To initiate the rare-cell *sc*WB workflow, we dilute blood samples in phosphate buffer saline (PBS) and process the cells using the Vortex chip technology (Fig. 1, Step i). The ∼300 μl sample effluent is then deposited into a ∼700 μl mesofluidic polydimethylsiloxane (PDMS) chamber mated onto the top surface of the *sc*WB device. Once inside the mesofluidic chamber, we stain with Hoechst 33342 all enriched cells to identify large, nucleated cells using both bright-field and epifluorescence microscopy. The microtransfer of a single putative cancer cell follows previously established protocols by Hannemann *et al*.^41^ and others. Briefly, although monitoring under epifluorescence microscopy, each large nucleated cell was picked individually using a precision Eppendorf Transferman micromanipulator and transferred to a microwell at a target occupancy of one cancer cell per microwell (Fig. 1, Step ii and Supplementary Fig. 1). Strongly Hoechst-stained cells with diameters larger than an average WBC were selected to exclude freely suspended red blood cells and WBCs. Nevertheless, cells associated with the putative cancer cell could also be transferred. Co-transfer of CTCs and CTC-associated cells (clusters), which includes WBCs and other cell types, offers potential utility, as the presence of associated cells is hypothesized to have an impact on patient prognosis^42^.

Next, the rare-cell *sc*WB assay is initiated. Each cell is subjected to in-microwell chemical lysis (15 s at 55 °C, 0.5% SDS, 0.1% Triton X-100 and 0.25% sodium deoxycholate (Na-DOC)) and an electric potential is applied across the *sc*WB device for single-cell PAGE (25 s, *E*=40 V cm^−1^, sieving matrix in an 8%T PA gel). After PAGE, proteins are immobilized in the gel using photo-blotting (45 s ultraviolet exposure, PA copolymerized with 100 mM *N*-[3-[(3-Benzoylphenyl)formamido]propyl] methacrylamide)^43^, followed by several buffer exchanges and antibody-probing cycles (Fig. 1, Step iii). The strong covalent immobilization of protein onto the gel, achieved by photo-blotting, enables multiple in-gel re-probing rounds, here demonstrated for a diverse panel of protein targets for each CTC (Fig. 1, Step iv).

### Rare-cell *sc*WB validation for CTC-specific protein profile

To perform assay development and validation, we utilized healthy donor blood spiked with cancer cell lines representing three major and diverse breast cancer subtypes: triple-negative (BT-20), ER+ (MCF7) and HER2+ (SK-BR-3). To create cell suspensions, we released plated cells using trypsin and spiked 300–600 cells from one cell line at a time into a 2 ml vial of healthy donor blood diluted 10 × in PBS. The antibody probes used bind to the cytoplasmic domains of EGFR and HER2. In accordance with literature^44^, our fluorescence-activated cell sorting-based validation (Supplementary Fig. 2) demonstrate negligible effect of trypsin release on the detection of surface EpCAM using the antibody probe selected here. The single-cell PAGE assay is operated under denaturing but non-reducing conditions, which retains intact EpCAM epitopes^45^ suitable for immunoreagent-based detection. After spiking the cancer cells into healthy blood, the samples were purified in the same manner as patient-derived CTCs.

**Figure 2:**
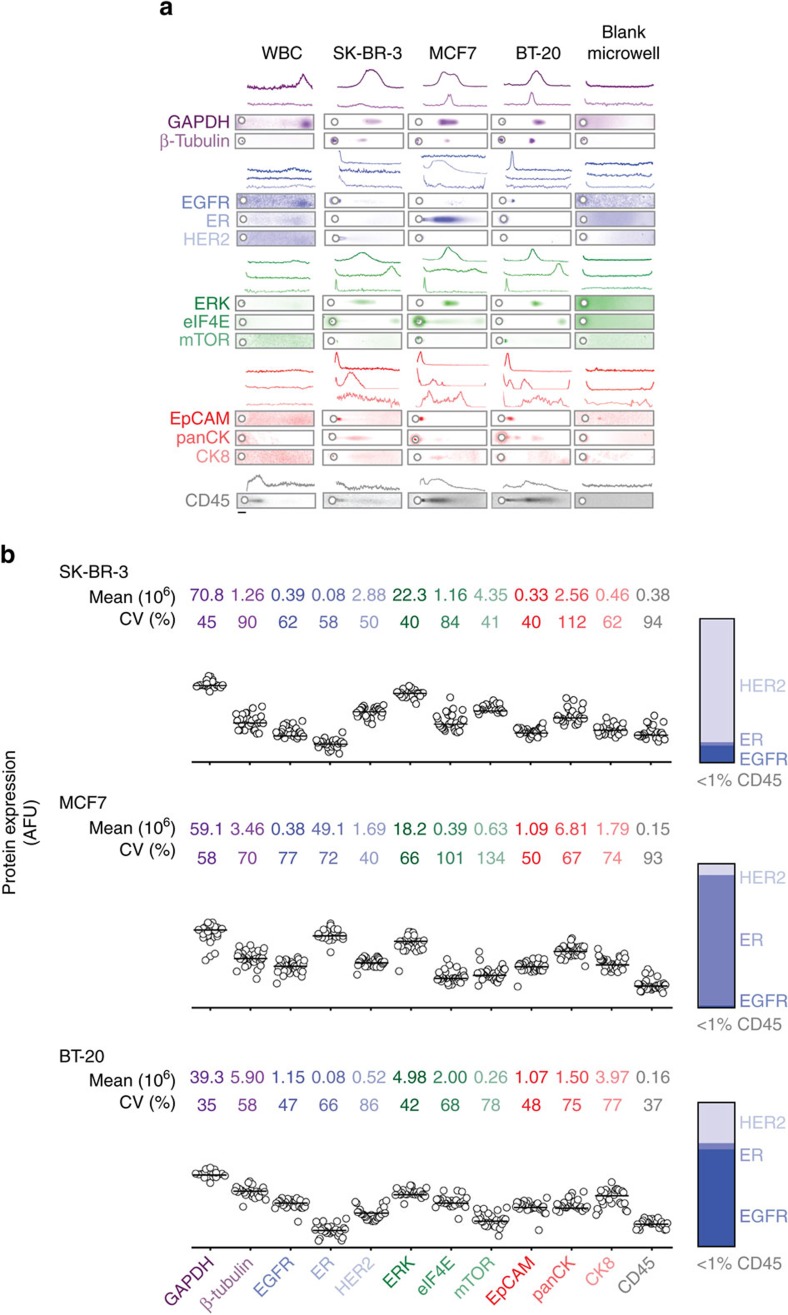
The rare-cell *sc*WB measures 12 unique protein targets in single cells from three cancer subtypes. (**a**) Fluorescence micrographs and intensity plots from rare-cell *sc*WB handling and analysis of healthy blood samples, each spiked with a cancer subtype: EGFR+(BT-20), ER+(MCF7) and HER2+(SK-BR-3). Negative controls include analysis of WBCs only and blank microwells (that is, devoid of a cancer cell). Protein panel comprises the following: control and housekeeping proteins (GAPDH and β-tubulin), oncoproteins (HER2, ER and EGFR), signalling proteins (ERK, eIF4E and mTOR), common CTC classifiers (EpCAM, panCK and CK8) and a WBC indicator (CD45). Scale bar, 50 μm. (**b**) Comparative protein expression for each cancer cell (BT-20: *n*=27; SK-BR-3: *n*=27; MCF7: *n*=35), with mean and CV noted for each marker. Protein expression is graphed using a log-scale. Ranked oncoprotein expression for each cell line agrees with cancer subtype. Less than 1% of total protein signal is attributable to CD45.

We first sought to assess the feasibility of the rare-cell workflow. A total of 50–110 min was sufficient for breast cancer cell isolation from the blood (20 min), followed by cell selection and seating of a single putative cancer cell into each microwell (30–90 min). Given a 30–90 min duration for cell selection and transfer, we measured one-cell-to-one-microwell transfers with: 42±19 s.d. BT-20 transfers (*n*=3 devices), 31±6 s.d. MCF7 transfers (*n*=3 devices), and 39±7 s.d. SK-BR-3 transfers (*n*=3 devices) achieved. Once microwells were populated with individual cancer cells, the *sc*WB assay proceeded as follows: in-microwell chemical cell lysis (seconds), protein PAGE and photo-blotting (immobilization; 2 min), subsequent in-gel primary (2 h) and secondary (1 h) antibody probing with one wash step after each probing cycle (1 h per wash), collection of fluorescence data (45 min) and an overnight (12 h) antibody stripping step for subsequent re-probing of additional proteins (Fig. 2a).

As baseline for gauging biological variation among patient-derived CTCs, we established sources of assay technical variation. First, we considered device-to-device variability by performing technical PAGE replicates on a solution of fluorescently labeled purified protein (an ovalbumin (OVA) standard), in a manner similar to that employed in our previous *sc*WB characterization studies^30^. We observed acceptably low device-to-device variation in both the protein peak width (*w*, coefficient of variation (CV)=14%, *n*=3 devices) and electromigration distance (*L*, peak maximum location at PAGE completion, CV=5%; Supplementary Fig. 3). In corollary studies of each of the three breast cancer cell lines, we confirmed that *sc*WB measurements of protein expression were comparable between unique *sc*WB devices (Mann–Whitney *U*-test, *P*>0.05 for BT-20, SK-BR-3 and MCF7; Supplementary Information).

Second, we considered variability in protein expression measurements by benchmarking (i) epifluorescence imaging of intact, green fluorescent protein (GFP)-expressing MCF7 cells (MCF7-GFP cells) seated in microwells against (ii) *sc*WB analysis of immunoprobed GFP from those same cells (Supplementary Fig. 3). First, we observed an appreciable linear correlation between the GFP signal measured in intact MCF7-GFP cells and the corresponding immunoprobed GFP signal (*R*^2^=0.83, Supplementary Fig. 3). Second, we estimated the technical variation threshold that distinguishes biological from technical variation, as has been utilized in both proteomics and single-cell RNA-sequencing analyses^46^,^47^. Defining detectable biological variability in protein expression as variation that is >3 s.d. from the mean protein expression CV yields a technical variation cutoff of 32.4% (GFP-MCF7 area under the curve (AUC) mean CV+3 s.d., where mean CV=11.0% and s.d.=7.1%; Supplementary Fig. 3).

To establish the selectivity of the protein panel targets (Supplementary Table 1) and to assess blood matrix effects, we compared the rare-cell *sc*WB analyses of spiked cell lines with two negative controls as follows: (i) microwells occupied only by WBCs and (ii) microwells containing no cancer cells (that is, ‘blank' microwells). The rare-cell *sc*WB reported positive signal for cancer markers for all spiked cell lines (BT-20, SK-BR-3 and MCF7) when a large, nucleated putative cancer cell was microtransferred into each microwell (Fig. 2a). In no instance did microscopy indicate transfer of more than one cancer cell. Further, in conjunction with microscopy, other researchers have shown microtransfer to be effective for isolation of individual cancer cells^41^,^48^,^49^. In negative controls, rare-cell *sc*WB analyses of pure WBC populations reported only CD45 and GAPDH signal, with no measurable signal from any cancer-specific protein target, thus establishing co-expression of CD45 and GAPDH, and negative cancer marker response as selective for WBCs. When microwells were empty in the spiked cancer cell studies (‘blank' wells), the rare-cell *sc*WB did not detect CD45 or GAPDH. In assessing potential ‘cross-talk' between proximal microwells, we did not observe detectable protein in empty microwells proximal to cancer marker-positive cell-laden microwells, with the exception of low-level EpCAM background. Thus, the validation study suggests that (i) the cancer markers are selective for putative cancer cells (as expected from the literature) and (ii) the protein panel allows the rare-cell *sc*WB to distinguish between cancer cells and WBCs.

As a corollary observation regarding possible association of WBCs with cancer cells, rare-cell *sc*WB analysis detected CD45 signal at the level of <1% of the total protein signal in putative cancer cells (Fig. 2b). Perhaps more surprising is the observation that the cancer cell-associated CD45 signal is not detected in ‘blank' microwells proximal to cancer cell-laden microwells. The observation indicates that WBC sedimentation into neighbouring microwells does not occur often, perhaps in light of the 10^4^- to 10^5^-fold reduction in WBCs during cancer cell isolation^32^. Consequently, the CD45 signal detected concomitant with the cancer panel-positive *sc*WBs suggests the possibility of WBCs associated with the putative cancer cells and not simply dissociated (or lysed) WBCs as ‘background' in the cancer cell-enriched blood. Recent transcriptomic data suggests that WBCs physically associate (‘cluster') with circulating tumour cells^42^. As is relevant to emerging clinical indicators of prognosis, the observation corroborates the utility of the rare-cell *sc*WB to capture both single CTCs and perhaps also CTCs associated with other cells (although association mechanisms are not currently understood).

### Rare-cell *sc*WB enables detailed assessment of key proteins

After establishing the selectivity of the cancer markers for cancer cells from three distinct cell lines and establishing the technical variation in the assay performance, we sought to scrutinize the known molecular classifier of each cancer subtype by rare-cell *sc*WB and compare with the literature. For each nucleated cell seated in a microwell, we ranked the relative expression of the three oncoproteins considered here (HER2, ER and EGFR; Fig. 2b). We observed concordance between the molecular classifications reported in the literature^50^,^51^,^52^ and those measured by the *sc*WB (that is, high HER2 expression in SK-BR-3, high ER expression in MCF7 and moderate EGFR expression in BT-20; Fig. 2). Of note, the rare-cell *sc*WB detected EGFR protein in the MCF7 cell line, in accordance with literature reports that indicate non-zero, yet low-level EGFR expression in these cells^53^. The primary molecular classification marker exhibited higher variability than other classification markers in two of the cancer cell lines (HER2: *σ*_SK-BR-3_^2^=2.08 × 10^12^, *n*=27, ER: *σ*_MCF7_^2^=1.24 × 10^15^, *n*=35; EGFR: *σ*_BT-20_^2^=3.15 × 10^11^, *n*=27).

For all protein panel targets across all three breast cancer cell lines (Fig. 2b), we measured variability in protein expression that exceeded the 32.4% technical variation cutoff; thus, we attribute the observed variation in protein expression to biological differences. We observed moderate-to-low variation in EpCAM expression for all cells, when compared with other proteins within each cell line (Supplementary Table 2). However, EpCAM expression in MCF7 and BT-20 cells had a wider distribution than that observed in the SK-BR-3 cell lines (*σ*_MCF7_^2^=3.00 × 10^11^; *σ*_SK-BR-3_^2^=1.80 × 10^10^; *σ*_BT-20_^2^=2.72 × 10^11^). For surface proteins known to be minimally or not expressed in each cancer subtype, we observed minimal variability in expression among each population (for example, ER in BT-20 and SK-BR-3).

Next, we applied the rare-cell *sc*WB and Spearman's rank-order correlation to study intracellular signalling proteins across all three cell subtypes. We measured appreciable correlation between the proteins ERK and GAPDH (*r*_BT-20_=0.759, *r*_MCF7_=0.782, *r*_SK-BR-3_=0.722, *P*<0.01), and the protein β-tubulin with both ERK (*r*_BT-20_=0.611, *r*_MCF7_=0.473, *r*_SK-BR-3_=0.667, *P*<0.01) and GAPDH (*r*_BT-20_=0.682, *r*_MCF7_=0.776, *r*_SK-BR-3_=0.825, *P*<0.01; Supplementary Table 3). Then, within each cancer subtype, we identified specific correlations in expression levels. First, in the MCF7 cell line, we observed considerable correlation between EpCAM and ER (*r*=0.670, *P*<0.01), as well as between EpCAM and panCK (*r*=0.616, *P*<0.01). We found panCK correlated with ER (*r*=0.662, *P*<0.01). Second, in the SK-BR-3 cells, we observed higher mTOR heterogeneity than the other cell lines (*σ*_SK-BR-3_^2^=3.25 × 10^12^, as compared with *σ*_BT-20_^2^=5.10 × 10^10^ and *σ*_MCF7_^2^=7.65 × 10^11^), with correlation between eIF4E and β-tubulin (*r*=0.728, *P*<0.01), as well as panCK and eIF4E (*r*=0.815, *P*<0.01). Third, BT-20 cell-to-cell expression of ERK was less variable (*σ*_ERK_^2^=4.51 × 10^12^) than observed in the MCF7 (*σ*_ERK_^2^=1.45 × 10^14^) and SK-BR-3 (*σ*_ERK_^2^=7.88 × 10^13^) cell lines (Supplementary Tables 2 and 3). Further, within the BT-20 population, EpCAM correlated well with eIF4E (*r*=0.692, *P*<0.01), mTOR (*r*=0.674, *P*<0.01) and ERK (*r*=0.663, *P*<0.01). Thus, although individual cells may be categorized by the same molecular subtype, differences in downstream signalling were evident within each population. As compared with the <5-plex protein multiplexing that is typical in immunocytochemistry^54^, numerous and even unexpected relationships may be identified by deeper profiling, such as that provided by 8- to 12-plex *sc*WB analysis of cancer cells.

### Lysis and electrophoresis optimization for CTC analysis

To the patient-derived CTCs, we applied lysis conditions optimized for the three cell lines (Patient 1; 15s lysis at 55 °C using 0.5% SDS, 0.1% Triton X-100, 0.25% Na-DOC). Interestingly, we observed inadequate electrophoretic injection of CTC lysate from the microwell into the *sc*WB sieving gel, which suggested incomplete solubilization lysis and solubilization. The observation suggests differences in the lysis phenotype between the three cell lines and the patient-derived CTCs^55^.

**Figure 3:**
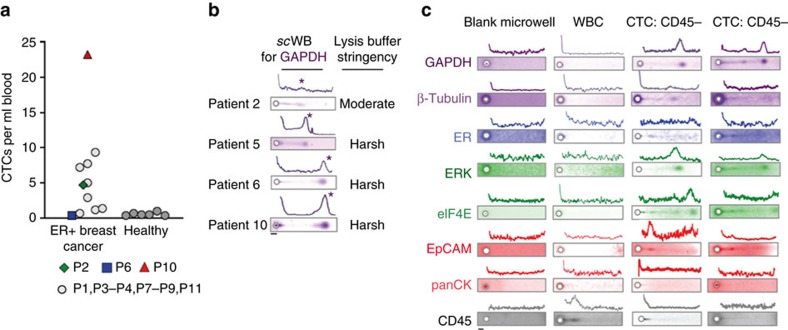
Optimization of the rare-cell *sc*WB for direct protein measurement in patient-derived CTCs. (**a**) CTC counts normalized to the blood volume processed by the isolation tool. CTC count for metastatic ER+ breast cancer patients (*n*=12): 0.33–23.25 CTCs per ml; CTC count for age-matched healthy donors (*n*=6): 0.33–1.00 CTCs per ml. CTC threshold was set by mean+2 s.d. from healthy donor data at 1.06 CTCs per ml, with 81.8% of the breast cancer patients classified as positive for CTCs. Enumeration for Patient 5 was not possible, as the sample was consumed by the *sc*WB. (**b**) Fluorescence micrographs and intensity plots from rare-cell *sc*WB analysis of GAPDH in CTCs from Patients 2, 5, 6 and 10 of CTC lysis conditions. Asterisks mark GAPDH peaks. (**c**) Fluorescence micrographs and intensity plots from rare-cell *sc*WB handling and analysis of representative patient-derived CTCs using nomenclature from Fig. 2a. Micrographs of rare-cell *sc*WB of patient-derived CTCs in representative cases where CD45 was not detected (CD45−). Scale bars, 50 μm.

Given the hardy, lysis-resistant CTC phenotype observed, we systematically varied lysis buffer composition, duration and temperature, and assessed CTC lysis and subsequent protein electrophoresis. As background, ionic detergents (SDS, Na-DOC) solubilize the cell membrane and denature proteins, whereas non-ionic detergents and lysis duration enhance membrane solubilization^56^. Buffer temperature enhances both solubilization and denaturation. Increasing lysis duration and elevating buffer temperature present trade-offs, as increasing each factor may yield more effective cell lysis but concomitantly increase diffusive losses of single-cell lysate out of the protein-permeable microwell^31^. Doubling the SDS concentration yielded satisfactory PAGE for cells from Patient 2 (Patient 2; 15s lysis at 55 °C using 1.0% SDS, 0.25% Triton X-100, 0.25% Na-DOC) by visual inspection of GAPDH injection dispersion (peak width=303 μm for 1 cell) (Fig. 3). However, CTCs derived from two subsequent patients (Patients 3 and 4) showed unacceptably high GAPDH injection dispersion, thus suggesting patient-to-patient differences in the lysis phenotype of CTCs.

Based on these findings, we further increased detergent concentrations above the critical micelle concentrations (CMC_SDS_=0.17%; CMC_Triton-X_=0.016%) (Pierce Protein Methods. Detergents for Cell Lysis and Protein Extraction. Protein Biology Resource Library) with Triton X-100 expected to reduce the SDS CMC, thus enhancing formation of mixed micelles that aid cell lysis and protein denaturation^57^,^58^. We extended the lysis duration and elevated the lysis buffer temperature (that is, Triton X-100 at 1%, Na-DOC at 0.5% and lysis buffer duration of 20–25 s at 60–65 °C). The increased temperature aids in reducing the CMC. Under these stringent lysis conditions, we observed successful lysis and separations of CTCs from Patients 5, 6 and 10.

### Patient-derived CTCs have a distinct biophysical phenotype

After optimization of the rare-cell *sc*WB assay on the cancer cell lines spiked into healthy blood (positive control) and WBC samples (negative control), we applied the workflow to CTCs isolated from 12 metastatic breast cancer patients. For each patient, two tubes of blood were processed, one for CTC enumeration (6 ml with 0.33 to 23.25 CTCs per ml) and another for *sc*WB (6–10 ml; Fig. 3a and Supplementary Table 4). The number of CTCs counted after immunostaining did not match to the number of CTCs analysed by rare-cell *sc*WB, as each measurement was performed on a unique blood fraction with no guarantee of a matched number of CTCs in each. The rare-cell *sc*WB was suitable for protein analysis of CTCs from patients with both low (Patient 6) and high (Patient 10) CTC counts.

Rare-cell *sc*WB yielded a unique measurement set that established a biophysical ‘lysis phenotype' for each individual CTC (Fig. 3b and Supplementary Fig. 4). Under stringent lysis conditions, we observed CTC lysis and successful electrophoretic injection of single-CTC lysate for an eight-component protein panel (Fig. 3c). Qualitatively, we observed generally low electromigration of EpCAM and ER into the PAGE gel, which we attribute to the presence of multimers (for example, 39 kDa EpCAM forms dimers and tetramers^59^) and secondary/tertiary structure (for example, ER), as noted by others^60^. Taken together, the stringent lysis conditions were applied rare-cell *sc*WB analyses of patient-derived CTCs from Patients 5–11.

### Multiplexed rare-cell *sc*WB analysis of patient-derived CTCs

**Figure 4:**
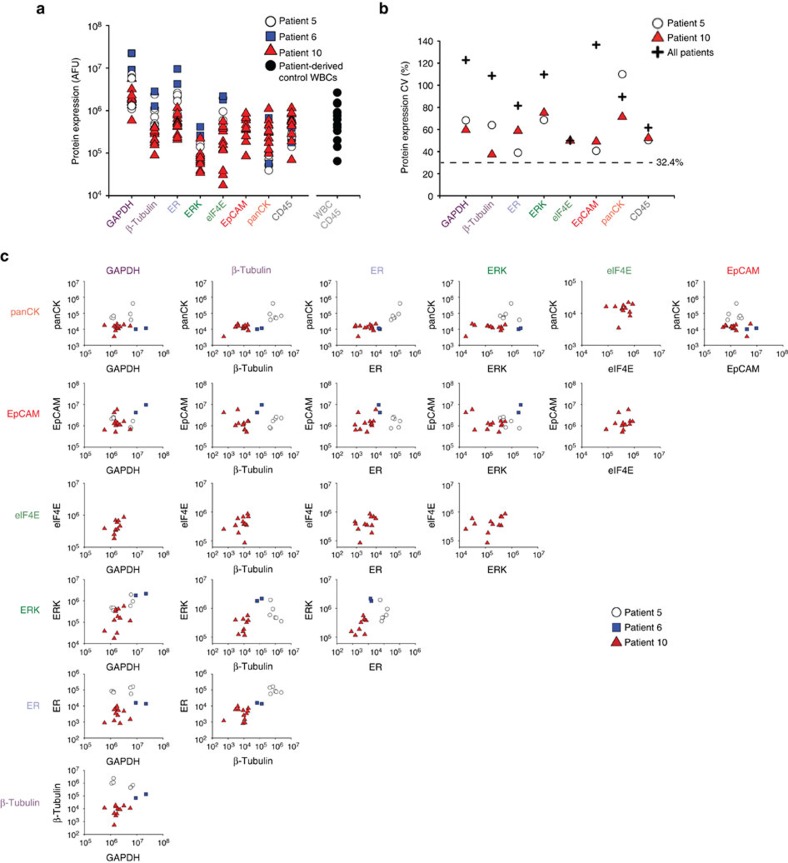
*sc*WB profiles for eight proteins in each individual CTC derived from three ER positive metastatic breast cancer patients. (**a**) Expression for each protein marker and each patient-derived CTC, with comparison with CD45 levels from *sc*WB analyses of pure WBC controls. (**b**) CVs for protein expression (AUC) from the patient-derived CTCs. Dashed line indicates the threshold in protein expression variation established using GFP-expressing MCF7 cells (see Supplementary Fig. 3). (**c**) Biaxial plots report protein expression for all markers for each patient-derived CTC from Patients 5 (*n*=6), 6 (*n*=2) and 10 (*n*=12).

Building on knowledge gained from breast cancer cell line spiking studies, we next sought to profile the protein panel in patient-derived CTCs using the rare-cell *sc*WB. We assayed a total of 12 unique protein targets in each putative CTC through 6 rounds of stripping and re-probing^30^. Of the 12 targets, we identified a sub-set of 8 detectable proteins in at least 1 CTC from patient samples 5, 6 and 10 (GAPDH, β-tubulin, panCK, ERK, EpCAM, ER, eIF4E and low expression CD45; Fig. 4a and Supplementary Fig. 5). The same protein panel was also applied to patient-derived WBCs, which showed measurable signal for CD45 and GAPDH only (Supplementary Fig. 6). Twenty patient-derived cells in total were isolated using size-based enrichment followed by physical selection of only the large, nucleated cells (Hoechst 33342) aided by both bright-field and epifluorescence microscopy. All of these selected cells reported positive for the tumour markers via *sc*WB. A seven-cell subset of the tumour marker-positive cells reported CD45+ by *sc*WB, suggesting the presence of both a cancer cell and associated leukocyte(s) in 35% of cells analysed. A 13-cell subset of the tumour marker-positive cells reported CD45− by *sc*WB, thus strongly suggesting CTC origin alone in 65% of the cells analysed.

Protein target expression was normalized by microwell occupancy, which was one CTC per microwell with the rare-cell handling workflow described. We attribute the observed CTC-to-CTC variation in protein expression levels to biological differences, as all protein panel targets had variability that exceeded the technical variation cutoff (Fig. 4b). In EpCAM expression, we observed a wider distribution among the CTCs, as compared with the ER+ MCF7 cells (*σ*_CTC_^2^=4.33 × 10^12^, *n*=20; MCF7: *σ*_MCF7_^2^=3.00 × 10^11^, *n*=35, Levene's test: *P*<0.05). This CTC EpCAM expression heterogeneity underscores the challenge for immunocapture-based enrichment. In GAPDH expression, the patient-derived CTCs exhibited a larger CV than the MCF7 cells, suggesting that the patient-derived CTCs have higher variation in GAPDH (CV_CTC_=123%, CV_MCF7_=57.9%, Levene's test: *P*<0.05). Conversely, in ER protein expression the patient-derived CTCs exhibited a narrower distribution than the MCF7 cells (*σ*_CTC_^2^=8.20 × 10^9^, *n*=20; *σ*_MCF7_^2^=1.24 × 10^15^, *n*=35; Levene's test: *P*<0.05). Taken together, these findings point to potential limitations of using cancer cell lines as models for patient-derived CTCs.

To further contextualize CTC-to-CTC variation in protein expression, we compared each patient-derived CTC with all other patient-derived CTCs for Patients 5, 6 and 10 (Fig. 4c). In contrast to the cell line spiking experiments, with patient-derived CTCs we observed low variability in GAPDH expression between Patients 5, 6 and 10 (Levene's test: *P*>0.05). With regards to Patients 5 and 10, we observed high CVs for panCK (CV_P5_=110% CV_P10_=71%, *P*<0.05) and ERK (CV_P5_=69%; CV_P10_=75%, *P*<0.05). In accordance with our breast cancer cell line observations, the ER+ breast cancer patient-derived CTCs also exhibited correlation between ERK and GAPDH (*r*_CTCs_=0.579, *P*<0.01; *n*=20) and between the pairs ERK with β-tubulin (*r*_CTCs_=0.691, *P*<0.01; *n*=20) and EpCAM with β-tubulin (*r*_CTCs_=0.6, *P*<0.01; *n*=20).

Among the patient-derived CTCs, we detected no statistically significant outlier CTCs (F-test)—with regards to exceptionally high or low protein expression—when target expression was normalized to the number of CTCs analysed in each *sc*WB (that is, one CTC analysed per microwell). However, the GAPDH expression suggests two distinct CTC sub-populations (Fig. 4c), which was confirmed with a F-test comparing a one-population model and a two-subpopulation model for the data resulting in an F-statistic of 3.89 and a *P*-value of 0.035 (degrees of freedom: (1,4)), thus suggesting two possible populations of GAPDH expression.

## Discussion

The rare-cell *sc*WB expands the repertoire of available single-cell analysis tools, an area where direct measurement of multiple protein targets in each CTC is hindered by reliance on single-stage immunoassays. Expanding the number of proteins profiled in a single CTC may aid identification of CTCs that do not fit contemporary taxonomies in light of the rapidly evolving understanding of CTC biology and individual responses to therapy. Notably, both existing and emerging precision cancer therapies are targeting proteins^35^,^39^; thus, monitoring multiple upregulated proteins in blood-derived CTCs may inform therapeutic selection to maximize the benefit to each specific patient at each specific time point. Consequently, we measure eight surface and intracellular proteins with single-CTC resolution using a microfluidic targeted proteomics tool, the rare-cell *sc*WB. Complementary single-cell resolution technologies such as flow cytometry and mass cytometry find analyses of such rare cells challenging, owing to sampling losses (that is, mass cytometry samples up to ∼30% of the initial cell population)^61^. The *sc*WB leverages short distances and timescales to rapidly complete lysis, PAGE and protein blotting of proteins in single CTCs. The careful control of sample and analysis affords the unique option to forgo normalization by protein housekeeping proteins and directly assign protein levels on a *per CTC* basis.

To first validate and then optimize the rare-cell *sc*WB tool, we spiked healthy blood with cells from each of three breast cancer cell lines to model three major molecular breast cancer classifications. We enriched for the spiked cancer cells and assayed via *sc*WB. Workflow durations of 50–110 min are well within the timeframe of other CTC processing and analysis methods^62^. *sc*WB classification of each cell line subtype agreed with expected molecular classification, but with heterogeneity in surface and intracellular protein expression detected at the single-cell level within the cells of each cell line. Tool validation studies further established the conditions needed for cell lysis, electrophoresis, blotting and probing, as well as established levels of technical variability. Rapid unit operations (lysis, electrophoresis and immobilization to the gel scaffold) and quick handling minimized dilution and diffusive losses of the cellular contents before assay completion, allowing the assay to maintain high local protein concentrations, even if local protein copy numbers are low (for example, ∼30,000 molecules per cell)^30^.

Pilot testing of the rare-cell *sc*WB collection–isolation–analysis workflow on blood from ER+ breast cancer patients yielded CTC enumeration data paired with *sc*WB protein analyses (Supplementary Table 4). The *sc*WB revealed a robust physical CTC phenotype resistant to the chemical lysis conditions that were optimized on three breast cancer cell lines. We attribute the hardy CTC phenotype to differences in cell type, handling and microenvironment as compared with breast cancer cell lines. For example, inclusion of fetal bovine solution (FBS) in the cell line culture medium is known to introduce a high concentration of lipids; thus, the lipid membrane composition of a cell grown in FBS would not necessarily be representative of primary cells^63^. Observation of the separation quality aided systematic optimization of lysis conditions suitable for a subset of eight protein targets, while minimizing protein losses. Experiments performed with our final lysis protocol suggest patient-to-patient variation in the physical CTC phenotype, thus mirroring observations even in cultured CTCs, where doubling time, point mutations and drug sensitivity vary within a CTC population^64^.

The precision and detection sensitivity of the rare-cell scWB enables direct analysis of individual, patient-derived CTCs, obviating the need for post-isolation cell culture. Variation in cell state and response is proposed to be a driver of cancer progression and therapy resistance^65^. The rare-cell *sc*WB identified differences between the CTC protein expression profiles, including both wider EpCAM expression ranges in CTCs (as compared with the three cell lines) and sub-populations of CTCs having statistically distinct GAPDH expression levels. Expansion of our multiplexed protein measurements with label-free CTC isolation to characterize additional subpopulations including cancer stem cell-like CTCs^66^ and CTCs undergoing EMT^67^ are of continuing interest. Looking forward to longitudinal studies, inclusion of protein levels in CTC phenotype could make CTC taxonomy more precise, as CTCs are thought to navigate away from the primary tumour perhaps diverging at the proteomic level^54^,^68^.

Based on our observations on CD45 expression in rare-cell *sc*WB analyses of large, nucleated cells, we see next steps developing in a manner that parallels the development of CTC enrichment tools optimized for ‘CTC cluster' analyses, so that future rare-cell western blotting work is focusing on specific assay optimization for CTC clusters, as both the CTC isolation tool^32^ and the microtransfer protocol^42^ used here have been shown to be suitable for CTC cluster handling.

Important to longitudinal studies, the *sc*WB is archivable^30^, which allows later-date profiling of new targets of interest with previously analysed single-CTC lysates. CTC lysates are covalently immobilized to the PA gel layer on the *sc*WB device, a stable linkage compatible with long-term storage for retrospective CTC analysis. Although chemical fixation of CTCs also yields biospecimens suitable for long-term storage and retrospective studies, cell fixation is incompatible with stripping procedures and suffers from several pre-analytical variables including the following: epitope disfigurement during fixation especially changes in posttranslational modifications, loss of antigenicity owing to over- and under-fixation, and sample degradation with aging. In addition, analytical variables including target cross-reactivity with moderate specificity antibodies, lab-to-lab variation and qualitative but not quantitative analyses, all have an impact on final outcomes. Although the rare-cell *sc*WB requires further long-term storage performance characterization and larger-scale patient studies, the targeted proteomics tool introduced here presents a promising approach to multiplexed, archival protein analysis of single CTCs with direct relevance to longitudinal studies. Even longer term, the microfluidic form factor of the assay may find utility in low-resource settings, especially with further engineering integration of the cell purification and handling fluidics, as is underway.

## Methods

### SU-8 and PA gel fabrication

Fabrication of the SU-8 microwell mold master and PA gels was performed as described previously^29^,^30^. The cell line and CTC experiments used an 8%T PA gel, with arrays of 50 μm diameter and 60 μm-deep microwells. All PA gels were chemically polymerized with 0.08% APS and 0.08% TEMED. A separate SU-8 master was created to fabricate the PDMS mesofluidic insert used to localize large volumes (∼700 μl) of enriched cells over the microwell array in the PA gel layer. The insert dimensions were 37.5 mm × 50 mm with an opening comprising the volume reservoir of 20 mm × 30 mm. PDMS polymer base and curing agent were mixed (ratio 10:1), degassed under vacuum, poured over the insert master and cured for 2 h at 70 °C.

### Cell lines

To validate the rare-cell *sc*WB, we acquired healthy donor blood and spiked with cell lines representing three major breast cancer subtypes: triple-negative (BT-20), ER+ (MCF7) and HER2+(SK-BR-3). BT-20, MCF7 and SK-BR-3 cells were obtained from the American Type Culture Collection and authenticated using short tandem repeat analysis (Promega). All cell lines tested negative for mycoplasma. BT-20 (ER−/PR−/HER2−) was maintained in Eagle's minimal essential medium supplemented with 1% penicillin/streptomycin and 10% FBS. MCF7 (ER+/PR−/HER2−) was maintained in RPMI 1640 supplemented with 1% penicillin/streptomycin, 0.01 mg ml^−1^ insulin (Invitrogen) and 10% FBS. SK-BR-3 (ER−/PR−/HER2+) was maintained in McCoy's 5A supplemented with 1% penicillin/streptomycin and 10% FBS.

A GFP-expressing MCF7 cell line, used to determine technical variation, was obtained from the American Type Culture Collection and authenticated using short tandem repeat analysis (Promega), and tested negative for mycoplasma. The cell line was maintained in RPMI 1640 supplemented with 1% penicillin/streptomycin, 0.01 mg ml^−1^ insulin (Invitrogen) and 10% FBS. All cell lines were cultured in an incubator held at 37 °C under 5% CO_2_ and tested for mycoplasma contamination.

### Patient recruitment and blood donation

Twelve patients with advanced breast cancer were recruited, with informed consent, according to a protocol approved by the Institutional Review Board (Stanford IRB 350–Panel 3–Protocol 5630) from the Department of Oncology at the Stanford School of Medicine. Blood was drawn in EDTA BD tubes, stored at room temperature and processed within 5 h after collection.

### Rare-cell enrichment from blood samples

A previously reported and commercially available microfluidic tool (Vortex Biosciences, Menlo Park, CA) was used for label-free isolation of circulating cancer cells in both the cell line spiking and cancer patient blood experiments. For spiking experiments, cells were dissociated with 1.5 ml of 0.25% trypsin (Life Technologies) and incubated in full media at room temperature to recover from exposure to trypsin. Cells were immediately spiked into healthy blood samples diluted 10 × with PBS and enriched through the Vortex HT chip, which uses microscale vortices to retain large cancer cells, while allowing smaller blood cells to exit as effluent^32^. The microfluidic device was first primed with PBS. Then, the diluted blood sample was processed through the Vortex HT chip (8 ml min^−1^) followed by a wash step with PBS to remove contaminating red blood cells and WBCs (8 ml min^−1^). Stopping the flow dissipates the vortices and releases the cancer cells from the microscale reservoirs for direct deposition on the top surface of the *sc*WB platform. The enriched volume was ∼300 μl and was contained by a mesofluidic PDMS insert that sits atop the *sc*WB. For the spiking experiments, 300–600 cells from 1 cell line were spiked into 1 ml healthy donor blood and processed using the Vortex chip. For patient blood experiments, the cells isolated in the vortices were directly collected into the mesofluidic PDMS insert seated on top of the *sc*WB PA gel for cell positioning into microwells. For both cell line spiking and patient-derived cell experiments, a volume of blood was reserved for subsequent red blood cell lysis to perform control experiments with WBCs.

### Preparation of WBCs

WBCs were prepared by lysing the red blood cells with Buffer EL (Qiagen). Briefly, 0.5 ml of whole blood was combined with 2.5 ml of Buffer EL (Qiagen). The tube was inverted several times and incubated for 10–15 min at room temperature. After centrifugation at 228 *g* for 5 min at room temperature, the supernatant was discarded. The pellet was re-suspended with 2.5 ml of Buffer EL and the process repeated. Finally, the WBCs were washed once with 1 ml of Buffer EL, pelleted and re-suspended in 0.5 ml PBS.

### *sc*WB protocol

The *sc*WB assay comprises six steps. The *sc*WB device utilizes microwells cast into a thin layer of a photoactive PA gel seated on microscope glass slide. Once aliquoted into the mesofluidic insert, cell nuclei were stained (Hoechst 33342) to identify target cells, and a micromanipulator (Eppendorf Transferman) and aspiration (Eppendorf Cell Vario) manually positioned individual cells into each microwell. A combined lysis and electrophoresis buffer was poured directly onto the PA gel where the cells were lysed in-well and then subjected to PAGE (*E*=40 V cm^−1^). Lysis buffer was heated in a water bath and the temperature was recorded with a thermometer immediately before use. After the PAGE separation, proteins were immobilized in the gel via brief ultraviolet activation (Lightningcure, LC5 Hamamatsu) of benzophenone methacrylamide cross-linked into the PA gel. Immobilized proteins were probed in-gel by diffusing primary and then fluorescently labelled secondary antibody probes into the PA gel layer. A fluorescence microarray scanner (Genepix 4300A, Molecular Devices) equipped with four-laser lines (488, 532, 594 and 635) acquired fluorescence readout. Subsequent rounds of antibody stripping were performed for multiplexed protein analysis as detailed previously^29^,^30^. The *sc*WB assay can be completed within ∼20 h.

### Antibodies

Primary antibodies and fold dilutions against GAPDH (1:20, goat polyclonal antibody (pAb); SAB2500450, Sigma), β-tubulin (1:10, rabbit pAb; ab6046, Abcam), EpCAM (1:10, rabbit pAb; 3599, Cell Signaling), EGFR (1:10, mouse monoclonal antibody; 2322, Cell Signaling), ER (1:10, rabbit monoclonal antibody; RM-9101-S0, ThermoScientific), HER2 (1:10, mouse monoclonal antibody; MA513105, Pierce), ERK1/2 (1:10, rabbit monoclonal antibody; 4695, Cell Signaling), eIF4E (1:10, rabbit monoclonal antibody; 2067, Cell Signaling), mTOR (1:10, rabbit monoclonal antibody; 2983, Cell Signaling), panCK (1:10, rabbit pAb; Z0622, Dako) and CK8 (1:10, mouse monoclonal antibody; C5301, Sigma) were the immunoprobes in both breast cancer cell lines (BT-20, MCF7 and SK-BR-3) and patient-derived CTCs. For analysis of the MCF7-GFP cell line, an anti-GFP antibody (ab6673, Abcam) followed by anti-goat AlexaFluor 555-conjugated secondary antibody (A21432, Invitrogen) were used. Secondary antibodies to goat IgG pre-labelled with AlexaFluor 488 and 555 (A11055 and A21432), mouse IgG pre-labelled with AlexaFluor 488, 555 and 647 (A21202, A31570 and A31571), and rabbit IgG pre-labelled with AlexaFluor 488, 555 and 647 (A21206, A31572 and A31573) were used as prepared by the vendor (Invitrogen). All secondary antibodies were applied as a 1:20 dilution.

### Flow cytometry

Flow cytometry analysis was performed on MCF7 cells to ascertain the effects of enzymatic detachment on the EpCAM antigen. Briefly, MCF7 cells were detached from tissue culture plates either by trypsin-EDTA (0.25%, Gibco 25200072) or by EDTA alone (Ultrapure 0.5 M EDTA Gibco 15575020 diluted in PBS to 5 mM). Half the cells were labelled with anti-EpCAM–AlexaFluor488 (mouse, monoclonal antibody, 53-8326-42, eBioscience) and half were labelled with mouse IgG AlexaFluor488 (A21202) as an isotype control. For labelling, 2 × 10^6^ cells were resuspended in 100 μl of 3% BSA (Sigma A2058) in PBS containing antibody at a concentration of 1 μg ml^−1^ and incubated over ice for 30 min. Cells were washed three times in PBS, then resuspended in resuspension buffer (1% BSA, 5 mM EDTA in PBS) to prevent aggregation. Cells were analysed on a Guava flow cytometer (Millipore). A total of 10,000 events were collected per sample, four samples per experimental group (*n*=4) and data were compiled and analysed using FlowJo software.

### Reproducibility

To measure the run-to-run variation in PAGE performance, we assayed solutions of purified OVA protein (O34781, Thermo Fisher Scientific) pre-labelled with AlexaFluor 488 diluted in PBS to a final concentration of 1 μM. PA gels were incubated with 100 μl of OVA solution for 1 h, to allow partitioning of OVA into the microwells after which the *sc*WB protocol was implemented. To measure the run-to-run variation in rare-cell *sc*WB performance (including cell lysis), we performed technical replicates on two separate *sc*WB devices for two aliquots each from a suspension of each cell line (BT-20, SK-BR-3 and MCF7). Each cell suspension was pipetted on top of the *sc*WB device and gravity-settled into microwells with excess cells washed off using a solution of 1 × PBS. After completing the *sc*WB protocol, GAPDH expression levels were measured (Supplementary Fig. 3); statistical equivalence of the GAPDH expression distributions between the technical replicates was tested using the Mann–Whitney *U*-test (‘ranksum' function in MATLAB R2013A). Mann–Whitney *U*-test *P*-values were 0.1257, 0.7578 and 0.7815 for BT-20 (*n*=59 and 65), SK-BR-3 (*n*=34 and *n*=30) and MCF7 (*n*=42 and 40), respectively. The null hypothesis that the GAPDH protein expression distributions are equivalent across the technical replicates was supported.

### Threshold for technical variation

Using a model GFP-expressing MCF7 cell line, we compared variation in GFP expression levels obtained by (i) fluorescence imaging of whole cells with (ii) *sc*WB analysis of probed GFP from those same cells. After establishing correlation between the two modalities, we established a technical variation threshold as described in the Results section. To perform the analyses, a suspension of GFP-expressing MCF7 cells (∼1 million cells per ml in 1 × PBS) was pipetted onto the *sc*WB device and cells settled by passive gravity into microwells. Excess cells were washed off with 1 × PBS as described elsewhere^29^,^30^.

For whole-cell imaging, epifluorescence microscopy recorded GFP fluorescence from MCF7-GFP cells seated in microwells (Olympus IX71 inverted fluorescence microscope, Andor iXon+ EMCCD camera, X-cite Lumen Dynamics mercury excitation lamp, ASI motorized stage controlled in Metamorph software, Molecular Devices). Supplementary Fig. 3 reports representative whole-cell fluorescence images ( × 10 Olympus UPlanFLN, numerical aperture 0.45 objective, GFP filter set Chroma 49011 ET, a binning of 1 and an exposure time of 200 ms). For *sc*WB analysis, the protocol described was used after imaging with MCF7-GFP cells lysed (15 s in 55 °C RIPA-like lysis buffer (0.5% SDS, 0.25% Na-DOC, 0.1% Triton X-100 in 0.5 × Tris-glycine), followed by PAGE (20 s at 40 V cm^−1^), photo-blotting (45 s), antibody probing for GFP (1:10 dilutions of anti-GFP antibody in 1 × TBST with 5% BSA, 2 h), wash (30 min in 1 × TBST), secondary immunoprobing (1:10 anti-goat AlexaFluor 555-conjugated secondary antibody in 1 × TBST, 1 h), wash (30 min in 1 × TBST), rinsed in water and dried in a nitrogen stream. For whole-cell images, a fluorescence intensity profile was generated in the microwell region of interest in ImageJ and the AUC was determined. For *sc*WB peaks, the AUC for the immunoprobed GFP peak was calculated using the *sc*WB analysis protocol (Supplementary Fig. 3). Cells with similar (<5% variation) GFP AUC were binned and considered a homogeneous GFP-expressing sample, with a 1.27–3.37% difference in AUC from the lowest and highest GFP AUC of each bin observed. The technical variation cutoff was defined as 3 s.d. above the average CV of protein expression (for a 99.7% confidence interval).

### Data analysis and processing

Quantification of protein PAGE and probing used in-house MATLAB scripts as described in Kang *et al*.^29^ Band widths were characterized by Gaussian curve fitting in MATLAB (R2014b, Curve Fitting Toolbox) if the Gaussian had a *R*^2^-value>0.7. If *R*^2^-value was <0.7 for a marker, the integrated intensity for the region of interest was calculated.

### Statistical analyses

Multiple statistical analyses were performed to compare protein distribution, correlations and potential outliers. To determine significance between the different protein CVs observed, we performed a *t*-test statistic and used a permutation test to determine the *P*-values. The Levene's test was used to determine non-equivalence of the variance between the markers for each cell line (BT-20, SK-BR-3 and MCF7). To classify a group of CTCs as a sub-population based on GAPDH expression in Patient 5, an F-test for model selection was performed. Model 1 assumed one population exists and model 2 assumed two sub-populations exist. The F-test compares the two models with the null hypothesis, considering the data follows model 1 instead of model 2. When the *P*-value is <0.05, the null hypothesis can be rejected.

To detect correlation in protein expression between proteins, a Spearman's rank correlation was performed, as the correlation of protein expression between two proteins was expected to be monotonic but not necessarily linear. Two proteins in the panel were sequentially paired (Supplementary Table 3) to determine possible correlations. Only correlations with a *P*-value ⩽0.01 were considered significant.

### Data availability

The authors declare that all the data are available within the article file and its Supplementary Information or from the corresponding author upon reasonable request.

## Additional information

**How to cite this article:** Sinkala, E. *et al*. Profiling protein expression in circulating tumour cells using microfluidic western blotting. *Nat. Commun.* **8**, 14622 doi: 10.1038/ncomms14622 (2017).

**Publisher's note:** Springer Nature remains neutral with regard to jurisdictional claims in published maps and institutional affiliations.

## Supplementary Material

Supplementary Information Supplementary Figures and Supplementary Tables
