# Supplementary material for: Profiling protein expression in circulating tumour cells using microfluidic western blotting
Source: Nat Commun. 2017 Mar 23;8:14622. doi: 10.1038/ncomms14622 (PMC5376644; doi:10.1038/ncomms14622)
Supplement: Supplementary Information — Supplementary Figures and Supplementary Tables [file ncomms14622-s1.pdf]

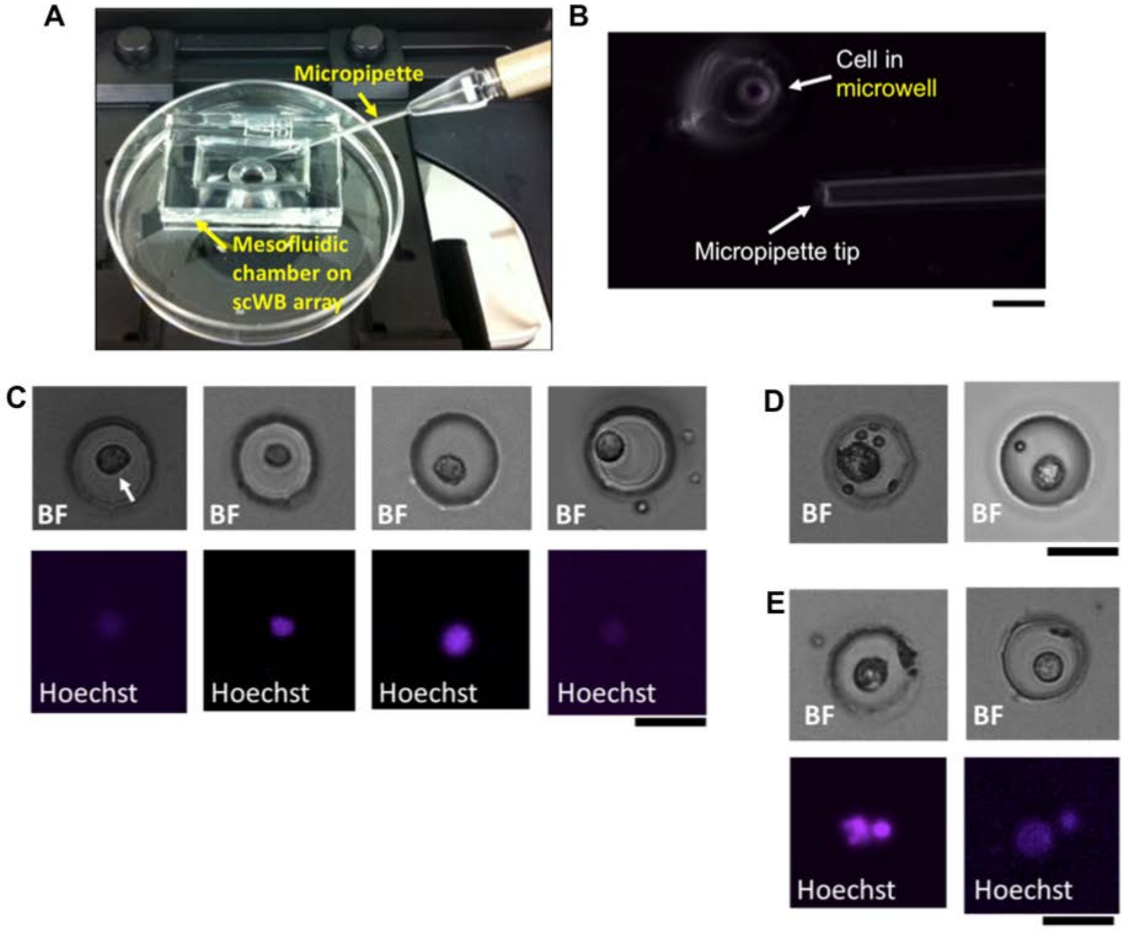

Supplementary Figure 1. Microwell single-cell occupancy is determined by visual inspection during microtransfer of large, nucleated cells from CTC-enriched, patient-derived blood samples. (**A**) Photograph of micropipette interfacing to a mesofluidic PDMS chamber mated on top of an *sc*WB array. The meso/microfluidic assembly is seated in a Petri dish on an inverted epi-fluorescence microscope stage. After visual inspection to determine microwell occupancy, the cells are subjected to *sc*WB for cancer cell and leukocyte protein markers. (**B**) Micrograph of micropipette tip proximal to a microwell housing a single cell. (**C**) Micrographs of microwells housing single CTCs after tumor-cell enrichment by the Vortex chip technology and microtransfer of each large, nucleated CTC into a microwell. Microwell occupancy of 1 putative CTC/microwell is determined using bright field (BF) and Hoechst nuclear stain (Hoechst) microscopy inspection. (**D**) Brightfield micrographs show individual, large putative CTCs seated in microwells containing associated (smaller) red blood cells. Associated leukocytes are confirmed during *sc*WB by positive CD45 signal. (**E**) Micrographs of microwells housing >1 Hoechst positive cell, as detected by visual inspection via BF and Hoechst and not included in single-CTC protein profiling analyses. Scale bars are 30 µm in (B)-(E). In (C)-(E), BF and Hoechst images are unique cells and not matched pairs.

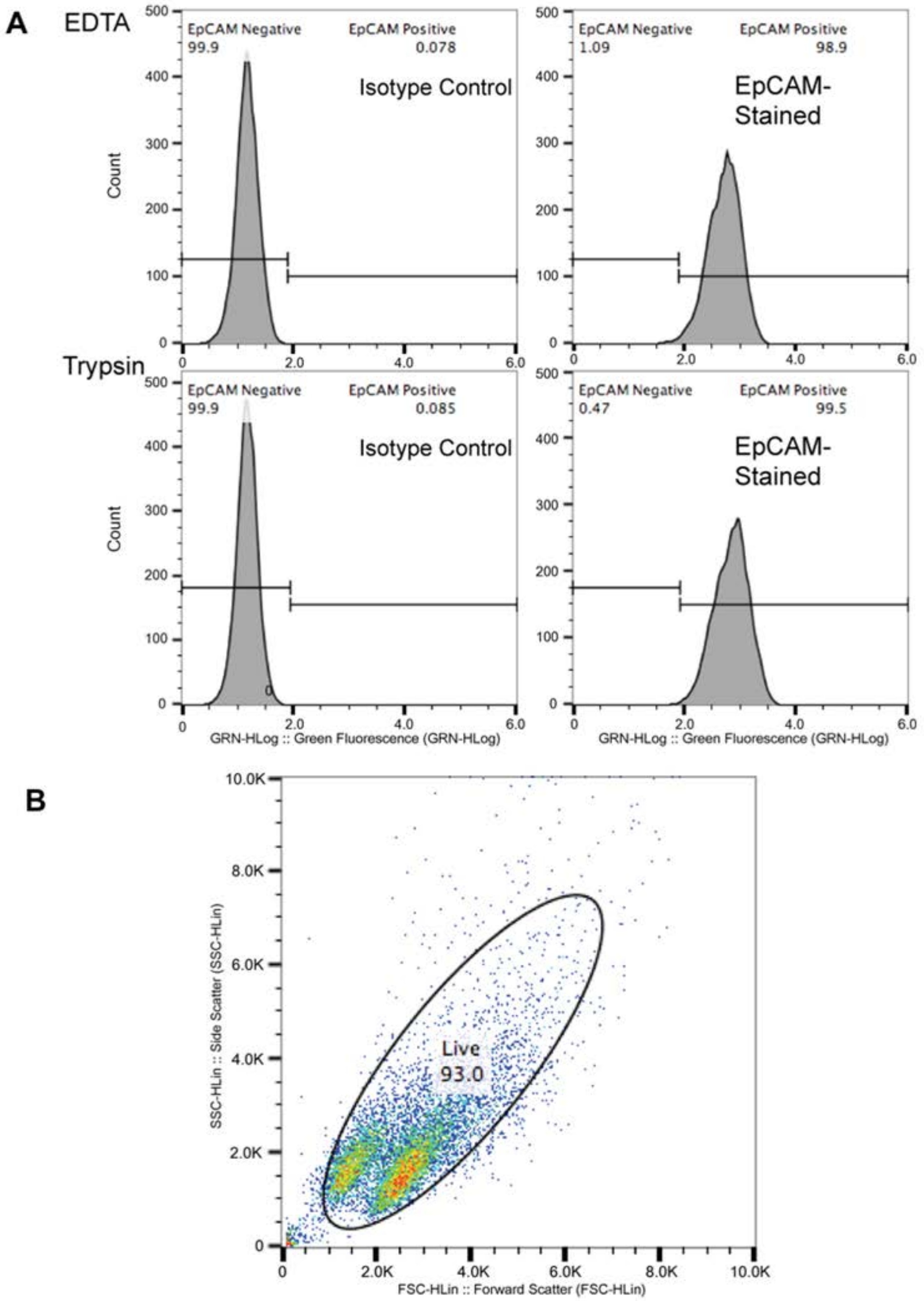

Supplementary Figure 2. Sensitivity of EpCAM antigen to enzymatic detachment. (**A**) Fluorescence intensity histograms of MCF7 cells labeled with isotype control (AlexaFluor488-mouse IgG) or anti-EpCAM-Alexa Fluor 488 antibody. Cells were detached either by 5mM EDTA (top) or trypsin-EDTA (bottom). In both cases, >98% of labeled cells were positive for EpCAM, using the isotype controls for EpCAM-negative gating (99±0.06% for trypsin/EDTA and 98±0.05% for EDTA, n=4 for both groups). (**B**) Forward scatter vs. side scatter plot of the cells detached by Trypsin-EDTA and stained with EpCAM, showing the gating strategy for live MCF7 cells.

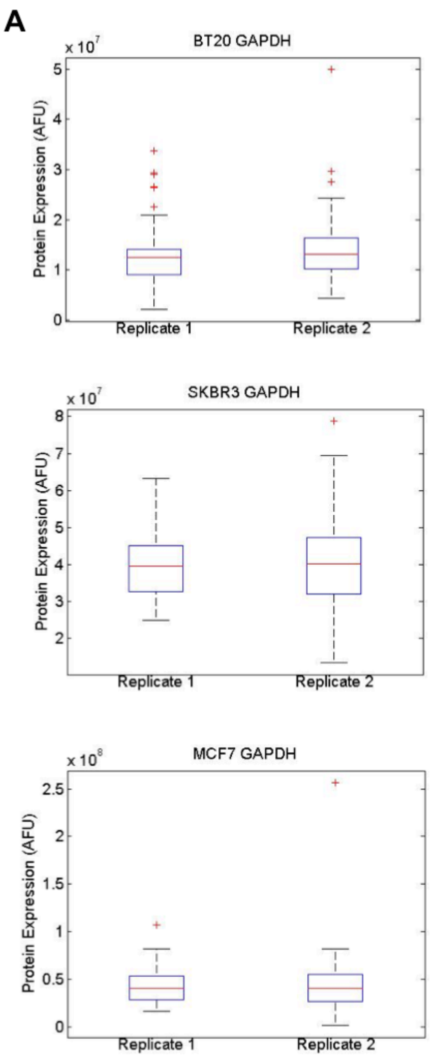

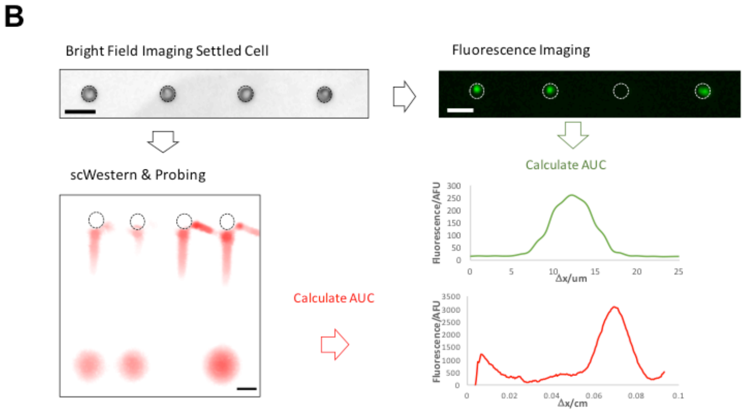

Cell	GFP AUC	Probed AUC	% Difference GFP	CV Probed (%)
1	166	95046	2.99	10.0
2	170	77774		
3	171	88965		
4	275	120137	1.27	5.5
5	275	132753		
6	278	132067		
7	367	145921	3.37	20.4
8	372	164309		
9	380	214914		
10	408	168585	3.14	15.9
11	414	153812		
12	421	208114		
13	1139	241967	1.63	3.3
14	1152	246009		
15	1157	230755		

CV Average	CV StDev	CV Threshold=avrg+3*stdev
11.0	7.1	**32.4**

Supplementary Figure 3. Inter- and intra- assay technical variation of protein expression in *sc*WB. (**A**) Comparison of GAPDH protein expression in scWB technical replicates using the indicated cell lines (scWB performed as described in the main text). Mann-Whitney U-test p-values were 0.1257, 0.7578 and 0.7815 for BT20 (n=59 and 65), SKBR3 (n=34 and n=30) and MCF7 (n=42 and 40) respectively, confirming the null hypothesis that the distributions of the technical replicates are equal. The red horizontal line represents the mean, and the upper and lower whiskers represent the 75th and 25th percentile respectively. (**B**) Brightfield and fluorescence images of MCF7-GFP cells in microwells, representative fluorescence micrograph and intensity profiles of GFP in the well and anti-GFP antibody probe signal, and table containing GFP and antibody probe signal values used to estimate intra-assay technical variation threshold. GFP-expressing cells are imaged by fluorescence microscopy in scWB wells prior to cell lysis and fluorescence of the cell is quantified. The *sc*WB is performed (as described in the main text) and GFP is detected with anti-GFP antibodies. Antibody probe signal (area-under-the curve, AUC) and coefficient of variation (CV) is quantified for cells that had <5% variation in intact GFP cell fluorescence. The technical variation threshold is calculated as the mean CV (11.0) plus three standard deviations (7.1, for a 99.7% confidence interval) yielding a CV threshold of 32.4%. Notably, all protein expression CVs from Fig. 2C (12 proteins, all three cell lines) are above the technical variation threshold, and thus we measure biological variation in the cell lines.

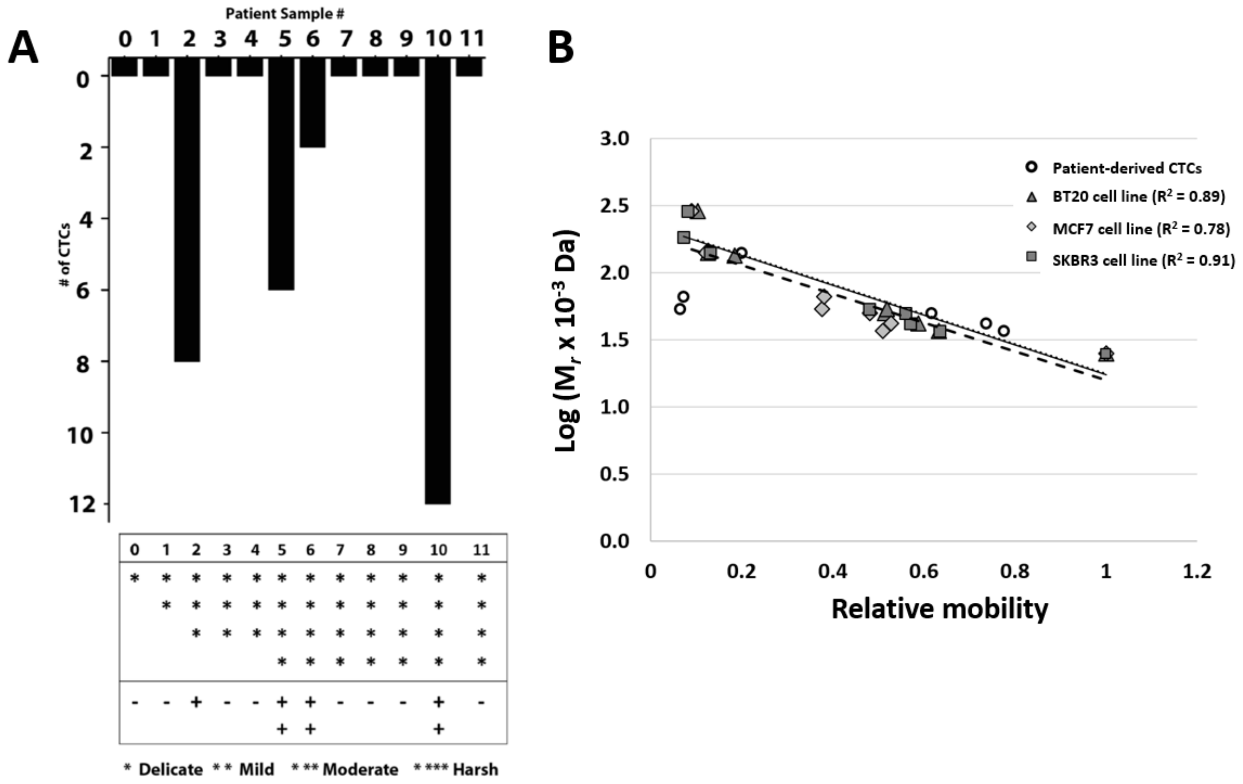

Supplementary Figure 4. Patient-derived CTCs present a more lysis-hardy biophysical phenotype than breast cancer cell lines as determined by rare-cell *sc*WB. (**A**) Lysis conditions of the CTCs processed in the *sc*WB. Differences in cell phenotype between breast cancer cell lines and CTCs required additional lysis optimization to assay proteins in CTCs. The lysis conditions tested were mild (10s lysis, 0.5% SDS, 0.1% Triton X-100, 0.25% Na-DOC at 45°C), moderate (15s lysis, 0.5% SDS, 0.1% Triton X-100, 0.25% Na-DOC at 55°C and 15s lysis, 1.0% SDS, 0.1% Triton X-100, 0.25% Na-DOC at 55°C) and harsh (20s lysis, 1.0% SDS, 1.0% Triton X-100, 0.5% Na-DOC at 60-65°C). Lysis effectiveness, the ability to resolve 1 or more proteins in a CTC, was rated for the CTCs assayed (e.g. No proteins resolved (poor/-), GAPDH only resolved (moderate/+), GAPDH and other proteins resolved (thorough/++)). (**B**) *sc*WB molecular mass calibration shows expected relationship between logarithm of molecular mass (M_r x 10^{-3}) and relative mobility (migration distance normalized to distance migrated for smallest protein target) for the 8-target protein sub-panel (eIF4e, EpCAM, GAPDH, ERK, βTUB, panCK, ER and mTOR, with EpCAM considered a tetramer). Comparison of calibration to patient-derived CTC proteins show as-expected electromigration by all targets, except ER and panCK (as discussed in the main text).

Patient 5

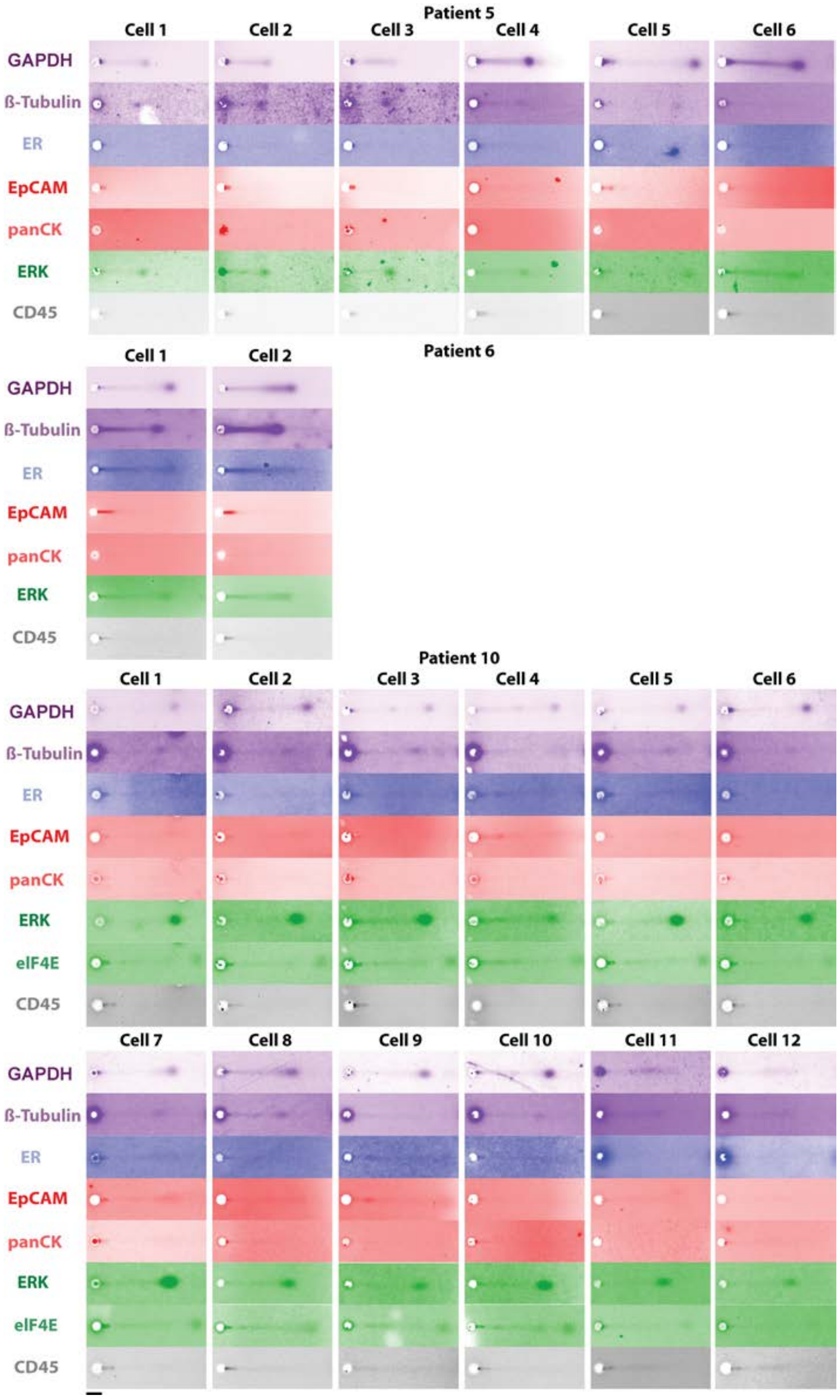

Supplementary Figure 5. *sc*WBs of patient-derived cells positive for the tumor protein profile. Large, nucleated cells isolated via the Vortex chip were individually deposited into microwells for subsequent *sc*WB analysis. The 8-plex protein profile was applied to each isolate cell. Scale bar is 100 μm.

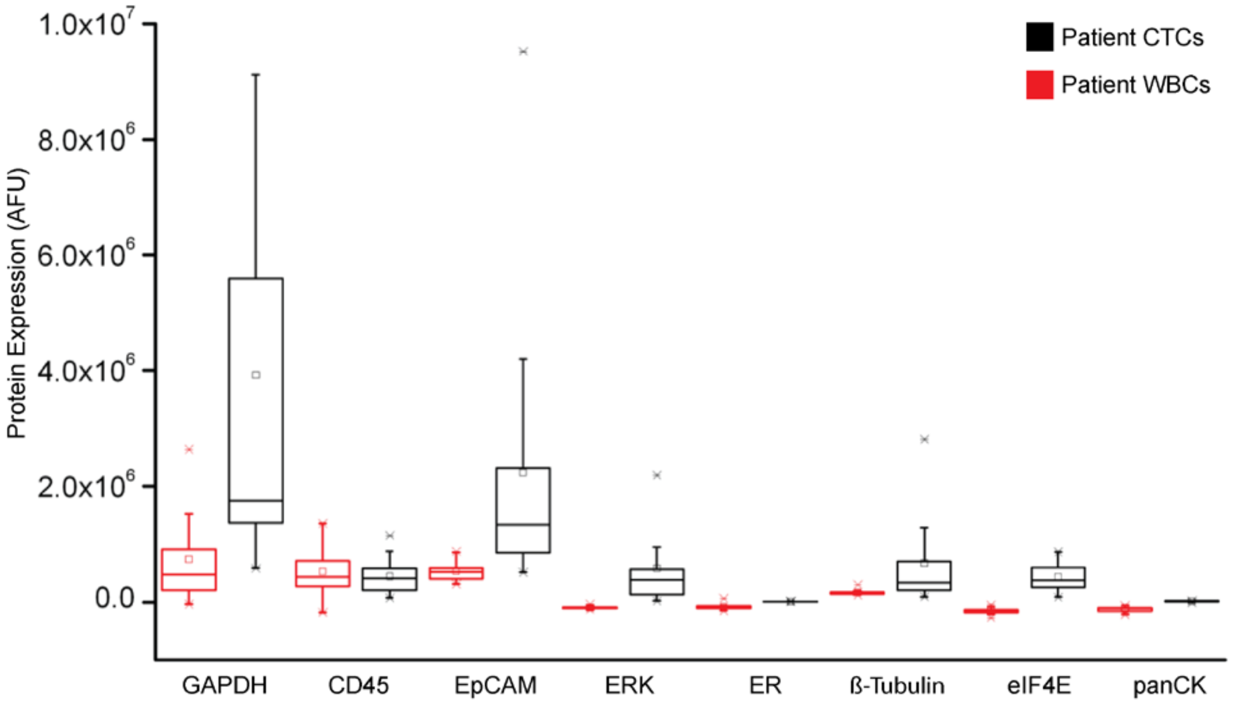

Supplementary Figure 6. Expression level distributions of the 8-protein panel for patient-derived CTCs and WBCs as determined by rare-cell *sc*WB. While both WBCs and CTCs show detectable CD45 and GAPDH levels, the WBCs show no other detectable protein from the panel assayed. For all the plots, the box represents the $25^{th}/75^{th}$ percentile, and the small square box represents the mean. Here, the whiskers are determined by the outermost data point the falls within the upper and lower inner fence (75th percentile ± interquartile range).

Classification	Protein	Molecular Weight (kDa)
Housekeeping proteins	GAPDH	37
	B-Tubulin	50
Oncoproteins	EGFR	134
	ER	66
	HER2	185
Signaling proteins	ERK1/2	42/44
	eiF4E	25
	mTOR	289
Common CTC classifiers	EpCAM	35
	panCK	40-67
	CK8	53
	CD45	147

Supplementary Table 1. Rare-cell *sc*WB protein panel includes both surface and intracellular proteins. Proteins and corresponding molecular masses are interrogated by the rare-cell scWB assay for both the cell lines (SK-BR-3, MCF7, BT20) and the patient-derived CTCs from ER+ metastatic breast cancer blood samples. Note EpCAM is thought to also exist in both dimer and tetramer forms, with electromigration measured by *sc*WB on cell line lysates consistent with the molecular mass of a tetramer.

SK-BR-3 n=27	GAPDH	β-Tubulin	EGFR	HER2	ER	ERK	eIF4E	mTOR	EpCAM	panCK	CK8	CD45
**Standard Deviation**	3.17E+07	1.12E+06	4.36E+05	1.44E+06	4.82E+04	8.87E+06	9.77E+05	1.80E+06	1.34E+05	2.86E+06	2.81E+05	3.58E+05
**Mean**	7.08E+07	1.26E+06	7.98E+05	2.88E+06	8.38E+04	2.23E+07	1.16E+06	4.35E+06	3.32E+05	2.56E+06	4.56E+05	3.80E+05
**Coefficient of Variation**	0.45	0.90	0.55	0.50	0.58	0.40	0.84	0.41	0.40	1.12	0.62	0.94
**Variance**	1.01E+15	1.26E+12	1.90E+11	2.08E+12	2.32E+09	7.88E+13	9.54E+11	3.25E+12	1.80E+10	8.19E+12	7.88E+10	1.28E+11

MCF7 n=35	GAPDH	β-Tubulin	EGFR	HER2	ER	ERK	eIF4E	mTOR	EpCAM	panCK	CK8	CD45
**Standard Deviation**	3.42E+07	2.43E+06	6.60E+05	6.76E+05	3.52E+07	1.21E+07	3.98E+05	8.75E+05	5.47E+05	4.59E+06	1.32E+06	1.42E+05
**Mean**	5.91E+07	3.46E+06	1.15E+06	1.69E+06	4.91E+07	1.82E+07	3.94E+05	6.29E+05	1.09E+06	6.81E+06	1.79E+06	1.53E+05
**Coefficient of Variation**	0.579	0.702	0.575	0.400	0.716	0.662	1.009	1.390	0.500	0.674	0.739	0.926
**Variance**	1.17E+15	5.90E+12	4.36E+11	4.57E+11	1.24E+15	1.45E+14	1.58E+11	7.65E+11	3.00E+11	2.11E+13	1.74E+12	2.00E+10

BT20 n=27	GAPDH	β-Tubulin	EGFR	HER2	ER	ERK	eIF4E	mTOR	EpCAM	panCK	CK8	CD45
**Standard Deviation**	1.45E+07	3.62E+06	5.66E+05	4.73E+05	5.61E+04	2.12E+06	1.37E+06	2.26E+05	5.22E+05	1.13E+06	3.10E+06	5.86E+04
**Mean**	4.02E+07	6.09E+06	1.43E+06	5.57E+05	8.32E+04	5.02E+06	2.01E+06	2.81E+05	1.10E+06	1.51E+06	3.79E+06	1.58E+05
**Coefficient of Variation**	0.362	0.594	0.396	0.849	0.674	0.423	0.681	0.805	0.475	0.750	0.819	0.372
**Variance**	2.11E+14	1.31E+13	3.20E+11	2.24E+11	3.15E+09	4.51E+12	1.87E+12	5.10E+10	2.72E+11	1.27E+12	9.62E+12	3.44E+09

Supplementary Table 2. Protein expression variance among breast cancer cell lines as determined by rare-cell *sc*WB.

CK8 vs GAPDH

	RANK	P-VALUE
SK-BR-3	0.721	2.21E-05
MCF7	0.447	7.13E-03
BT20	-0.382	4.52E-02

CK8 vs β-Tubulin

	RANK	P-VALUE
SK-BR-3	0.684	8.26E-05
MCF7	0.725	8.43E-07
BT20	-0.144	0.465

CK8 vs EGFR

	RANK	P-VALUE
SK-BR-3	0.726	1.82E-05
MCF7	0.536	9.15E-04
BT20	0.102	0.604

CK8 vs ER

	RANK	P-VALUE
SK-BR-3	0.263	0.185
MCF7	0.706	2.11E-06
BT20	-0.307	0.112

CK8 vs HER2

	RANK	P-VALUE
SK-BR-3	-0.471	1.31E-02
MCF7	0.272	0.114
BT20	-0.583	1.13E-03

CK8 vs ERK

	RANK	P-VALUE
SK-BR-3	0.642	3.10E-04
MCF7	0.122	0.484
BT20	-0.063	0.748

CK8 vs eIF4E

	RANK	P-VALUE
SK-BR-3	0.713	2.99E-05
MCF7	0.111	0.525
BT20	0.202	0.303

CK8 vs mTOR

	RANK	P-VALUE
SK-BR-3	0.444	2.02E-02
MCF7	0.388	2.13E-02
BT20	-0.210	0.284

CK8 vs EpCAM

	RANK	P-VALUE
SK-BR-3	0.045	0.823
MCF7	0.350	3.94E-02
BT20	0.115	0.560

CK8 vs panCK

	RANK	P-VALUE
SK-BR-3	0.734	1.29E-05
MCF7	0.425	1.10E-02
BT20	0.052	0.793

panCK vs GAPDH

	RANK	P-VALUE
SK-BR-3	0.695	5.79E-05
MCF7	0.463	5.06E-03
BT20	0.374	5.00E-02

panCK vs β-Tubulin

	RANK	P-VALUE
SK-BR-3	0.488	9.74E-03
MCF7	0.435	8.96E-03
BT20	0.270	0.165

panCK vs EGFR

	RANK	P-VALUE
SK-BR-3	0.574	1.75E-03
MCF7	0.205	0.237
BT20	0.114	0.564

panCK vs ER

	RANK	P-VALUE
SK-BR-3	0.025	0.901
MCF7	0.662	1.51E-05
BT20	0.332	8.41E-02

panCK vs HER2

	RANK	P-VALUE
SK-BR-3	-0.677	1.05E-04
MCF7	0.571	3.45E-04
BT20	-0.316	0.102

panCK vs ERK

	RANK	P-VALUE
SK-BR-3	0.481	1.11E-02
MCF7	0.125	0.476
BT20	0.650	1.83E-04

panCK vs eIF4E

	RANK	P-VALUE
SK-BR-3	0.815	2.26E-07
MCF7	0.245	0.155
BT20	0.256	0.189

panCK vs mTOR

	RANK	P-VALUE
SK-BR-3	0.359	6.59E-02
MCF7	0.295	8.54E-02
BT20	0.292	0.132

panCK vs EpCAM

	RANK	P-VALUE
SK-BR-3	0.071	0.723
MCF7	0.616	8.26E-05
BT20	0.350	6.81E-02

EpCAM vs GAPDH

	RANK	P-VALUE
SK-BR-3	0.322	0.102
MCF7	0.470	4.38E-03
BT20	0.551	2.40E-03

EpCAM vs β-Tubulin

	RANK	P-VALUE
SK-BR-3	0.347	7.58E-02
MCF7	0.469	4.52E-03
BT20	0.750	4.25E-06

EpCAM vs EGFR

	RANK	P-VALUE
SK-BR-3	0.016	0.935
MCF7	0.338	4.74E-02
BT20	0.276	0.154

EpCAM vs ER

	RANK	P-VALUE
SK-BR-3	0.437	2.28E-02
MCF7	0.670	1.08E-05
BT20	0.558	2.04E-03

EpCAM vs HER2

	RANK	P-VALUE
SK-BR-3	0.045	0.825
MCF7	0.511	1.70E-03
BT20	0.230	0.239

EpCAM vs ERK

	RANK	P-VALUE
SK-BR-3	0.106	0.598
MCF7	0.321	6.03E-02
BT20	0.663	1.21E-04

EpCAM vs eIF4E

	RANK	P-VALUE
SK-BR-3	0.102	0.613
MCF7	0.321	6.03E-02
BT20	0.692	4.46E-05

EpCAM vs mTOR

	RANK	P-VALUE
SK-BR-3	-0.112	0.579
MCF7	0.090	0.606
BT20	0.674	8.48E-05

mTOR vs GAPDH

	RANK	P-VALUE
SK-BR-3	0.288	0.146
MCF7	0.303	7.65E-02
BT20	0.733	9.20E-06

mTOR vs β-Tubulin

	RANK	P-VALUE
SK-BR-3	0.291	0.141
MCF7	0.462	5.25E-03
BT20	0.826	6.23E-08

mTOR vs EGFR

	RANK	P-VALUE
SK-BR-3	0.476	1.22E-02
MCF7	0.301	7.88E-02
BT20	0.344	7.28E-02

mTOR vs ER

	RANK	P-VALUE
SK-BR-3	0.003	0.988
MCF7	0.311	6.87E-02
BT20	0.648	1.96E-04

mTOR vs HER2

	RANK	P-VALUE
SK-BR-3	-0.162	0.418
MCF7	-0.043	0.806
BT20	0.574	1.42E-03

mTOR vs ERK

	RANK	P-VALUE
SK-BR-3	0.418	3.00E-02
MCF7	0.103	0.556
BT20	0.602	6.99E-04

mTOR vs eIF4E

	RANK	P-VALUE
SK-BR-3	0.198	0.323
MCF7	0.069	0.693
BT20	0.626	3.65E-04

eIF4E vs GAPDH

	RANK	P-VALUE
SK-BR-3	0.838	4.85E-08
MCF7	0.162	0.353
BT20	0.456	1.46E-02

eIF4E vs β-Tubulin

	RANK	P-VALUE
SK-BR-3	0.728	1.70E-05
MCF7	0.194	0.265
BT20	0.759	2.82E-06

eIF4E vs EGFR

	RANK	P-VALUE
SK-BR-3	0.561	2.33E-03
MCF7	0.171	0.325
BT20	0.344	7.28E-02

eIF4E vs ER

	RANK	P-VALUE
SK-BR-3	0.247	0.214
MCF7	0.273	0.112
BT20	0.392	3.89E-02

eIF4E vs HER2

	RANK	P-VALUE
SK-BR-3	-0.678	1.01E-04
MCF7	0.166	0.339
BT20	0.235	0.228

eIF4E vs ERK

	RANK	P-VALUE
SK-BR-3	0.548	3.07E-03
MCF7	-0.002	0.990
BT20	0.604	6.61E-04

ERK vs GAPDH

	RANK	P-VALUE
SK-BR-3	0.722	2.11E-05
MCF7	0.782	2.95E-08
BT20	0.759	2.82E-06

ERK vs β-Tubulin

	RANK	P-VALUE
SK-BR-3	0.667	1.46E-04
MCF7	0.473	4.10E-03
BT20	0.611	5.47E-04

ERK vs EGFR

	RANK	P-VALUE
SK-BR-3	0.694	6.04E-05
MCF7	0.574	3.10E-04
BT20	0.488	8.39E-03

ERK vs ER

	RANK	P-VALUE
SK-BR-3	0.330	9.31E-02
MCF7	0.527	1.15E-03
BT20	0.700	3.37E-05

ERK vs HER2

	RANK	P-VALUE
SK-BR-3	-0.261	0.189
MCF7	0.227	0.190
BT20	-0.007	0.971

HER2 vs GAPDH

	RANK	P-VALUE
SK-BR-3	-0.386	4.65E-02
MCF7	0.482	3.41E-03
BT20	0.330	8.69E-02

HER2 vs β-Tubulin

	RANK	P-VALUE
SK-BR-3	-0.296	0.134
MCF7	0.341	4.51E-02
BT20	0.608	5.97E-04

HER2 vs EGFR

	RANK	P-VALUE
SK-BR-3	-0.449	1.89E-02
MCF7	0.175	0.316
BT20	-0.031	0.877

HER2 vs ER

	RANK	P-VALUE
SK-BR-3	0.275	0.166
MCF7	0.582	2.47E-04
BT20	0.216	0.269

ER vs GAPDH

	RANK	P-VALUE
SK-BR-3	0.524	5.04E-03
MCF7	0.784	2.54E-08
BT20	0.799	3.41E-07

ER vs β-Tubulin

	RANK	P-VALUE
SK-BR-3	0.603	8.67E-04
MCF7	0.803	6.47E-09
BT20	0.540	3.00E-03

ER vs EGFR

	RANK	P-VALUE
SK-BR-3	0.089	0.661
MCF7	0.692	4.07E-06
BT20	0.276	0.154

EGFR vs GAPDH

	RANK	P-VALUE
SK-BR-3	0.154	6.76E-03
MCF7	0.643	3.12E-05
BT20	0.283	0.145

EGFR vs β-Tubulin

	RANK	P-VALUE
SK-BR-3	0.697	5.32E-05
MCF7	0.491	2.73E-03
BT20	0.288	0.137

β-Tubulin vs GAPDH

	RANK	P-VALUE
SK-BR-3	0.825	1.22E-07
MCF7	0.776	4.44E-08
BT20	0.682	6.42E-05

Supplementary Table 3. Protein expression correlations among the spiked cell lines as determined by rare-cell *sc*WB. The Spearman correlation and p-values were calculated for all protein pairs as described in the main text. Items highlighted in green represent significant correlations with p-values < 0.01.

No.	Type	Receptor status	Stage	Age	# CTC	# WBC	Vol. blood (mL)
0	Breast	ER-/PR-/HER2-	IV	58	186	427	8
1	Breast	ER-/PR+/HER2+	IV	60	46	113	6
2	Breast	ER+/PR+/HER2-	IV	51	28	106	6
3	Breast	ER+/PR+/HER2-	IV	78	17	130	6
4	Breast	ER+/PR+/HER2-	IV	66	30	93	6
5	Breast	ER+/PR+/HER2-	IV	61	N/A	N/A	N/A
6	Breast	ER+/PR+/HER2-	IV	56	2	84	6
7	Breast	ER-/PR-/HER2+	IV	61	8	168	7
8	Breast	ER+/PR+/HER2-	IV	68	8	65	6
9	Breast	ER+/PR+/HER2-	IV	71	4	1229	6
10	Breast	ER+/PR+/HER2-	IV	51	56	144	6
11	Breast	ER+/PR+/HER2-	IV	53	43	94	6
12	Healthy	N/A	N/A	62	2	403	6
13	Healthy	N/A	N/A	41	4	128	6
14	Healthy	N/A	N/A	57	2	56	4
15	Healthy	N/A	N/A	60	3	83	6
16	Healthy	N/A	N/A	69	6	104	6
17	Healthy	N/A	N/A	77	2	166	6

Supplementary Table 4. Patient and healthy donor information and CTC enumeration data. Patient samples 0-11 were processed both for scWB and enumeration by immunofluorescence (IF), while samples 12-17 were processed for enumeration only. Patient 5 was not enumerated as the total blood volume isolated was allocated to the *sc*WB assay workflow.
